# The nerve growth factor-delivered signals in prostate cancer and its associated microenvironment: when the dialogue replaces the monologue

**DOI:** 10.1186/s13578-023-01008-4

**Published:** 2023-03-20

**Authors:** Marzia Di Donato, Pia Giovannelli, Antimo Migliaccio, Gabriella Castoria

**Affiliations:** Department of Precision Medicine, University of Campania “L.Vanvitelli”, 80138 Naples, Italy

**Keywords:** Prostate cancer, Tumor microenvironment, Nerve-growth factor, Perineural invasion

## Abstract

Prostate cancer (PC) represents the most diagnosed and the second most lethal cancer in men worldwide. Its development and progression occur in concert with alterations in the surrounding tumor microenvironment (TME), made up of stromal cells and extracellular matrix (ECM) that dynamically interact with epithelial PC cells affecting their growth and invasiveness. PC cells, in turn, can functionally sculpt the TME through the secretion of various factors, including neurotrophins. Among them, the nerve growth factor (NGF) that is released by both epithelial PC cells and carcinoma-associated fibroblasts (CAFs) triggers the activation of various intracellular signaling cascades, thereby promoting the acquisition of a metastatic phenotype. After many years of investigation, it is indeed well established that aberrations and/or derangement of NGF signaling are involved not only in neurological disorders, but also in the pathogenesis of human proliferative diseases, including PC. Another key feature of cancer progression is the nerve outgrowth in TME and the concept of nerve dependence related to perineural invasion is currently emerging. NGF released by cancer cells can be a driver of tumor neurogenesis and nerves infiltrated in TME release neurotransmitters, which might stimulate the growth and sustainment of tumor cells.

In this review, we aim to provide a snapshot of NGF action in the interactions between TME, nerves and PC cells. Understanding the molecular basis of this dialogue might expand the arsenal of therapeutic strategies against this widespread disease.

## Introduction

Prostate cancer (PC) remains the most commonly diagnosed cancer in men worldwide [1] and therapeutic interventions are various, because of its heterogeneity [2]. At early stage, PC is prevalently treated with radical prostatectomy, brachytherapy, cryotherapy [3] and focal therapies [4, 5]. This cancer, however, frequently evolves towards a locally advanced disease. At this stage, the androgen deprivation therapy (ADT), associated or not with external beam radiotherapy [6], represents the backbone patient’s treatment. Nevertheless, oncologists still experience many frustrations because of the ineffectiveness of these approaches, mainly related to the therapy escape and disease progression. PC often becomes castration-resistant (CRPC), which can be metastatic or not [7]. Few approaches are actionable in these patients and the death toll remains paradoxically high, albeit the substantial improvements in early diagnosis and treatments [8].

The mechanisms related to PC progression and drug-resistance are still under intense investigation. Among them, the aberrant signaling mediated by sex steroid receptors (SRs), mainly the androgen receptor (AR), the derangement of growth factor signaling, the release of steroids, growth factors and chemokines by PC cells themselves or tumor microenvironment (TME) counterpart have been hypothesized [9–13]. Thus, the discovery of ‘druggable’ biomarkers has led to development of precision therapies. Beyond the selective AR modulators, such as abiraterone [14] and enzalutamide [15], whose efficacy is largely recognized, the list of currently available drugs includes accelerators of the AR degradation, neutralizing antibodies against PC cell specific proteins and tyrosine kinase receptors (TRKs) [16], chemical compounds inhibiting the poly(ADP-ribose) polymerase (PARP) [17–20] or the signaling effector activity and even small peptides perturbing the interactions between AR and various signaling effectors. Table 1 summarizes the new promising compounds studied in preclinical and clinical PC models.

**Table 1:** Emerging interventions and drugs used in clinical trials for the treatment of patients with advanced disease or in preclinical PC models

Target & mechanism of action	Intervention/treatment	Study name (If available)	Conditions/disease	Development stage	Study number
PARP inhibitor	Olaparib	PROfound Study	CRPC with BRCA1/2 mutations	Phase III clinical trial	NCT02987543
	Niraparib	Galahad	CRPC with DNA-Repair Anomalies	Phase II clinical trial	NCT02854436
	Rucaparib	TRIUMPH	Metastatic Hormone-Sensitive PC with Germline DNA Repair Gene Mutations	Phase III clinical trial	NCT03413995
	Rucaparib	ROAR	CRPC with BRCA gene alteration	Phase II clinical trial	NCT03533946)
	Talazoparib	TALAPRO-1	CRPC with DNA Repair Defects	Phase III clinical trial	NCT03148795
	Veliparib (ABT-888)	ABT-888	CRPC	Phase II clinical trial	NCT01085422
AR antagonists/inhibitors	JNJ-56021927(ARN-509; Apalutamide)	JNJ-56021927 effects on the Pharmacokinetics of Multiple Cytochrome P450 enzymes and transporter	CRPC	Phase I clinical trial	NCT02578797
	Proxalutamide (GT0918)	Proxalutamide (GT0918) in Subjects With CRPC	CRPC	Phase II clinical trial	NCT03899467
Hedgehog inhibitors	Vismodegib	A Pharmacodynamic Study of Vismodegib	CRPC With Accessible Metastatic Lesions for Tumor Biopsy	Phase I clinical trial	NCT01880437
anti-PDCD1 and -CTLA-4 (IgG1κ) antibodies	Nivolumab + ipilimumab	INSPIRE	immunogenic CRPC	Phase II clinical trial	NCT04717154
anti-PD-L1 Antibody + AR antagonist	Atezolizumab + Enzalutamide	IMbassador250	CRPC after Failure of an Androgen Synthesis Inhibitor	Phase III clinical trial	NCT03016312
anti-PD-1 + anti-IL-8 antibodies + GRH antagonist	Nivolumab + BMS-986253 + degareliz	MAGIC-8	Hormone-Sensitive PC	Phase I, Phase II clinical trials	NCT03689699
PD-1	Pembrolizumab	PERSEUS1	CRPC	Phase II clinical trial	NCT03506997
anti- IgG2 subtype binding and neutralizing (RANKL)	denosumab	Open-Label Access Protocol of Denosumab for Subjects With Advanced Cancer	Bone Metastases in Men With Hormone-Refractory PC	Phase III clinical trial	NCT01419717
AR Antagonist + a dual DNA-PK/TOR kinase (DNAPK/TORK) inhibitor	Enzalutamide + CC-115	A Phase 1b Study of Enzalutamide Plus CC-115 in Men With CRPC	PC; CRPC	Phase I clinical trial	NCT02833883
anti-Trop-2 antibody (hRS7) conjugated to SN-38 payload	IMMU-132: ADC based on a humanized	IMMU-132 on Second Generation AR-Directed Therapy	CRPC	Phase 2 clinical trial	NCT03725761
LSD1 inhibitors	GSK2870552			Preclinical study	
	GSK-LSD1			Preclinical study	
	RN-1			Preclinical study	[21] [22]
	SP-2509			Preclinical study	
Cytochrome P450 17A1inhibitor/Combinatorial approaches	Radium-223 Dichloride causing double-strand breaks in DNA + Abiraterone Acetate	ERA 223	CRPC	Phase III clinical trial	NCT02043678
	Abiraterone Acetate + Enzalutamide + Erdatifinib	Erdafitinib and Abiraterone Acetate or Enzalutamide in Treating Patients With Double Negative PC	Double negative PC	Phase II clinical trial	NCT03999515
	Abiraterone acetate (CB7630) + Prednisone/prednisolone	Abiraterone Acetate in CRPC Previously Treated With Docetaxel-Based Chemotherapy	CRPC	Phase III clinical trial	NCT00638690
EZH2 inhibitors	GSK126			Preclinical study	
	GSK343			Preclinical study	[23]
	GSK503			Preclinical study	
Aurora-A	MLN8237	Aurora Kinase A Inhibitor MLN8237 in Patients With Metastatic Castrate Resistant and Neuroendocrine PC	NEPC	Phase II clinical trial	NCT01799278
BRN2 inhibitor	BRN2i		Small cell PC	Preclinical studies	[24]
eIF2α/eIF2S1 Ser51 Phosphorylation inhibitor	ISRIB		Aggressive PC progression	Preclinical studies	[25]
Double-strand breaks in DNA + cyclical, high-dose testosterone administration	radium 223- Bipolar Androgen Therapy (BAT)	BAT-RAD	CRPC	Phase 2 clinical trial	NCT04704505
XPO-1 inhibitor	selinexor			Preclinical studies	[26]
hypoxic regions of solid tumors	selective hypoxia-activated prodrug Evofosfamide (TH-302)			Preclinical studies	[27]
AR/FlnA complex	Rh-2025 u			Preclinical studies in PC and PC-TME	[28]
AR/Src complex	S1 peptide			Preclinical studies in PC	[29]

*AR* Androgen receptor, *EZH2* Enhancer of zeste 2 polycomb repressive complex 2 subunit, *CRPC* metastatic castration resistant prostate cancer, *mTOR* mammalian target of rapamycin, *PARP* poly-ADP ribose polymerase, *PTEN* phosphatase and tensin homolog, *XPO-1* exportin 1, *RANKL* receptor activator NF kappa B ligand, *PCD1* Protocadherin 1, *PD-L1* Programmed death ligand 1, *IL-8* Interleukin 8, *PD-1* programmed cell death protein 1, *DNA-PK* DNA-dependent protein kinase, *ADC* Antibody Drug Conjugate, *Trop-2* Trophoblast cell surface antigen 2, *LSD1* lysine-specific demethylase 1A, *BRN2* Brain-Specific Homeobox/POU Domain Protein 2, *eIF2α/eIF2S1* Eukaryotic translation initiation factor 2 subunit 1, *Fln A* filamin A

Recent years have also seen very encouraging results from therapies that build on modulation of immune system, such as chimeric antigen receptor (CAR)T-cell therapy, vaccines, and immune-checkpoint inhibitors [30]. The currently used vaccine-based immunotherapeutic approaches are presented in Table 2.

**Table 2:** Novel vaccine-based immunotherapeutic approaches for PC patients

Type	Interventions	Study name	Condition/disease	Development stage	References or Study number
Bacteria-based cancer vaccine	JNJ-64041809 (ADU-741)	LADD Listeria monocytogenes bacteria	PC	Phase II clinical trial	NCT02625857
Active immunotherapy vaccine containing PSA to generate a T-cell response	PROSTVAC-V PROSTVAC-F GM-CSF	PROSTVAC-V/F ± GM-CSF	Asymptomatic or Minimally Symptomatic Metastatic CRPC	Phase III clinical trial	NCT01322490
autologous dendritic cells activated	DCVAC/PCa	VIABLE	CRPC	Phase III, IV clinical trial	NCT02107404
pTVG-HP + pTVG-AR is a plasmid DNA	pTVG-HP + pTVG-AR + Pembrolizumab	pTVG-HP DNA Vaccine With or Without pTVG-AR DNA Vaccine and Pembrolizumab	CRPC	Phase II clinical trial	NCT04090528

*LADD* Live attenuated double deleted, *PC* prostate cancer, *PSA* prostate-specific antigen, *GM-CSF* Granulocyte–macrophage colony-stimulating factor, *CRPC* castration resistant prostate cancer, *pTVG-HP* plasmid DNA, encoding the cDNA for human prostatic acid phosphatase (PAP), *AR* androgen receptor

Findings collected over the last decade have highlighted the role of growth factors, including insulin-like growth factor-1 (IGF-1) [31, 32], vascular endothelial growth factor (VEGF) [33], epidermal growth factor (EGF) [34, 35] and their dependent networks in PC pathogenesis and progression. Again, fibroblast growth factor (FGF) ligands control the development of prostate gland, as relevant levels of FGF2, FGF7 and FGF9 can be detected in normal prostate mesenchyme cells, while the cognate receptors, FGFRs, are expressed in secretory prostatic epithelium. Thus, FGF/FGFR signaling is necessary for development and homeostasis of the normal prostate gland. Derangement of FGF signaling is involved in PC development and progression and several findings have reported aberration of FGF/FGFR signaling throughout all the stages of PC, from the prostatic intraepithelial neoplasia (PIN) to carcinoma ‘in situ’, and then invasive and metastatic PC [36].

Despite these intense studies on the role of growth factors in prostate development and neoplastic transformation, the study of neurotrophins has been prevalently restrained to the field of neuronal biology and neuropathies, until it becomes almost neglected in human cancer. Many years have indeed passed from the discovery of nerve growth factor (NGF) in the early 1950’s [37] to the studies concerning its role in non-neuronal as well as cancer cells [38]. After many years of investigation, we now appreciate that aberrations and/or derangement of NGF signaling are involved in the pathogenesis of various human diseases, including cancers. Relevant to this manuscript are the increasing findings linking the NGF signaling to PC progression.

Here, we briefly report an update of these studies. The *pros* and *cons* of the role of NGF in PC will be presented, together with the evidence linking the NGF and its cognate receptors to the paracrine loop between PC and TME cells. These results have paved the way for unexpected concepts about the function of NGF signaling in PC cell plasticity and innervation. Beyond their impact in PC biology, the drivers of NGF-dependent signaling are currently targeted to improve the patient’s survival and the cancer-related pain.

## Neurotrophins and their receptors: structure and functions

**Fig. 1:**
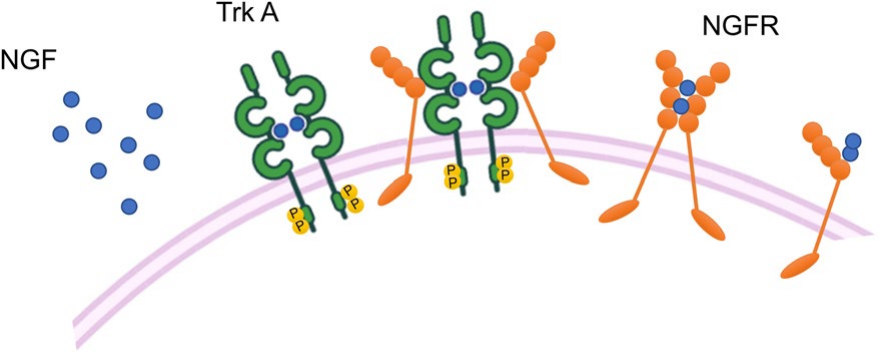
Activation of the high-affinity receptor TrkA and the low-affinity receptor NGFR by NGF. Left, TrkA activation by NGF leads to dimerization of the receptor and phosphorylation of different tyrosine residues in the intracellular domain, which in turn promote the formation of binding sites for signaling effectors. Right, NGFR binds NGF through the cysteine-rich repeats endowed within its extracellular domain. Middle, when TrkA and NGFR are co-expressed, NGFR might enhance the binding affinity of TrkA for NGF, either by increasing the NGF concentration at cell surface or modifying the Trk A conformation through allosteric interactions [47]

Until recently, the neurotrophin’s action has been almost exquisitely related to development of the nervous system. As such, neurotrophins have been distinguished from other growth factors, classically related to cell proliferation, for their ability to modulate neuronal differentiation [39]. This is, however, not the only difference. Neurotrophins are synthesized by neurons to act locally, but they can be also released by peripheral non-neuronal cells poised at considerable distance from the central nervous system [40]. To date, 4 neurotrophins have been identified in humans and named NGF, brain-derived neurotrophic factor (BDNF), neurotrophin-3 (NT-3) and neurotrophin-4/5 (NT-4/5; [41]. They derive from a unique ancestral gene, which has consequently split up on the different chromosomes 1, 11, 12 and 19 for NGF, BDNF, NT-3 and NT-4/5 genes, respectively. To achieve the complete mature form, the transcripts are then translated into precursors, the pro-neurotrophins that share the same molecular weight (almost 26 KDa). Pro-neurotrophins are cleaved by intracellular proteases at a highly conserved dibasic amino-acid cleavage site to generate the carboxy-terminal mature neurotrophins, migrating at 12–13 KDa [42]. The mature proteins give rise to stable, non-covalent dimers, whose action is mediated by the binding to membrane receptors, mainly the neurotrophin receptor p75NTR (also called NGF receptor; NGFR) and the neurotrophin tyrosine kinase receptor (Trk) family, which consists of three members, TrkA, B and C [43]. NGFR and TrkA specifically bind NGF (Fig. 1), while TrkB exhibits specific binding for BDNF and NT-4/5. Finally, TrkC shows specificity for NT-3 [44]. Pro-neurotrophins, however, can also bind NGFR or sortilin, a membrane glycoprotein member of the vacuolar protein sorting 10 protein (Vps10p) family, to execute opposing effects to neurotrophins in neuron development, damage-induced cell death and synaptic plasticity [45, 46].

The binding of neurotrophins to their specific receptors causes the dimerization of tyrosine kinase receptors, which then undergo auto-phosphorylation in intra-cytoplasmic tyrosine residues that serve as docking for many adapter proteins with the consequent signaling activation cascade. Of note, association of TrkA or TrkB with NGFR can regulate their binding to the cognate ligands [47], and neurotrophins (NGF, BDNF and NT-3) might also bind to α9β1 integrin, making it as a new neurotrophin receptor [48].

Several outstanding reviews have so far described the molecular mechanism engaged by neurotrophins in neuronal cells [44, 49, 50]. Nowadays, it is largely accepted that neurotrophin receptors mediate the activation of a *plaethora* of signaling effectors, including the phosphoinositide 3-kinase (PI3-K), the tyrosine kinase Src, the focal adhesion kinase (FAK) as well as the downstream mitogen-activated protein kinases (MAPKs) and protein kinase B (PKB or AKT) to transmit their survival and differentiation functions in the nervous system. Albeit initially related to neuronal cells, analysis of these pathways has been extended to non-neuronal cells, in both healthy and disease states, including inflammatory response, wound healing impairment, auto-immune disorders and dis-regulation of the nervous-endocrine axis, such as the alteration in insulin homeostasis involved in diabetes [41].

Intriguing findings on NGF signaling derangement have been discovered in the gender-related cancers, including PC. The next sections in this manuscript aim to concisely discuss these results, together with their impact on identification of biomarkers predictive of PC malignancy and drug-response.

## The NGF/NGF receptor signaling in PC

The role of neurotrophins in tumor biology is still under intense investigation. NGF receptors, initially discovered in gliomas, neuroblastomas and medulloblastoma [38], have been detected in many solid cancers, such as breast [51–54], lung [55, 56], colon [57], pancreas [58] and prostate [59]. Myelomas and lymphoid tumors are also sensitive to NGF and express high levels of NGF receptors [60]. However, conflicting findings have been reported about the role of NGF in human cancers. Studies in primary and cultured cancer cells have shown that NGF promotes differentiation and stops tumor progression [38]. These findings have been attributed to the differential expression of TrkA or NGFR, with the consequent acceleration of differentiation or proliferation, respectively. This dichotomy appears excessively simple and other aspects should be considered. Firstly, the cell outcome in response to NGF stimulation might depend on a pre-existent oncogenic stimulation. Again, the strength and duration of signaling might determine the cell fate [61]. At last, the availability of other signals produced in situ, such as steroid hormones might influence the response to NGF. Consistent with this latter hypothesis, several findings have pointed to the role of steroid/NGF connection in quite different systems [62–67], including PC cells [68].

NGF is produced by normal prostatic tissues and PC cells [69]. In the latter cells, it induces mitogenesis or apoptosis upon TrkA or NGFR binding, respectively [70]. Moreover, the neurotrophin receptors undergo significant changes during PC progression [71–74], as primary PC express both TrkA and NGFR, while losing NGFR during the progression towards a more aggressive phenotype. At last, NGFR is almost completely absent in metastatic PC [72–76], making the TrkA receptor as a driver of NGF signaling in aggressive PC.

As in neuronal cells, NGF triggers activation of various intracellular signaling cascades, controlled by Ras, PI3-K or PLC in PC cells representative of the different stages of the disease’s progression [77, 78]. Recent findings have supported a role for TrkA in NGF-elicited effects, as the receptor’s somatic knockdown impairs the NGF-triggered activation of the effectors leading to mitogenesis and invasion in aggressive PC cells [76]. Given these findings, inhibition of NGF circuit by specific drugs has attracted the interest of urologists and oncologists. Some years ago, it was shown that treatment of PC-derived cells with TrkA pharmacological inhibitors, such as K252a and CEP-701, reduces the NGF-elicited proliferation and fosters cell death [79, 80]. Therefore, the pan-Trk inhibitor, CEP-701 entered with great promises the phase-I clinical trial in PC patients [81], and phase-II clinical trials are still in progress in asymptomatic hormone-refractory PC patients. Noteworthy, CEP-701 inhibits other tyrosine kinase receptors, thus showing low specificity together with side-effects. Recently, orally available TrkA and pan-Trks inhibitors (VMD-928 and TQB3811 or ONOI-7579, respectively) or multiple kinase inhibitors (TSR-011; DS-6051b) have entered phase I clinical trials. The last update was posted in 2021 and it reported that these molecules are well tolerated and only induce gastrointestinal side-effects. Nevertheless, the studies are currently accruing in expansion cohorts. Particularly, the trial with VMD-928 is recruiting patients affected by thymic carcinoma, mesothelioma, squamous cell carcinoma of head and neck, squamous cell carcinoma of the lung, ovarian, hepatocellular and PC exhibiting TrkA overexpression. We recently reported that the TrkA inhibitor, GW441756 [82] perturbs the NGF-elicited signaling leading to proliferation and aggressiveness of various PC cells and impairs the size of PC-derived spheroids [76]. In addition to reinforcing the significance of TrkA targeting in aggressive PC, the findings in 3D models are very promising to assess the beneficial effects of novel compounds in preclinical drug screening. We are currently investigating the effect of small, modified peptides, perturbing the TrkA interactome in 3D models derived from PC cells at different degree of malignancy [28, 68–83], or patients (in preparation). It should be noted, however, that albeit the dissection of NGF action in PC cells, our preclinical studies left still pending the question of NGF signaling derangement in PC. It might be argued, for instance, that PC exhibits aberrations of NTRK (either NTRK1, or 2 or 3) genes, which yield to gene fusions. Nevertheless, these oncogenic alterations can be detected at high frequency (almost 90%) in rare cancer types (secretory breast carcinoma, mammary analogue secretory carcinoma, cellular or mixed congenital mesoblastic nephroma and infantile fibrosarcoma), but at very low frequency (< 1%) in other tumor types, including PC [84]. Thus, other mechanisms should be hypothesized to explain the NGF signaling derangement in PC.

A recent study reported that the E3 ubiquitin-ligase, TRAF4 ubiquitinates TrkA, thereby promoting its hyperactivation and lung metastasis. The finding that TRAF4 is highly expressed in metastatic PC not only expand the role of the ubiquitination system in human cancers, but also identifies TRAF4 as a new clinical biomarker, predictor of TrkA hyperactivation and PC aggressiveness [78]. These data also shed light on a new putative target for the treatment of aggressive PC. However, deregulation of NGF signaling in PC might be also due to the intersection between NGF and SRs, mainly the AR. Several years ago, it was reported that prolonged exposure to NGF induces the AR re-expression in androgen-insensitive DU145 cells [85], suggesting that NGF deeply impact the AR signaling, maybe through down-regulation of DNA methylation [86]. These results indicated that neurotrophins induce the receptor renaissance in AR-negative PC cells. Many years later, we showed that a reciprocal crosstalk between AR and TrkA occurs in neuronal and PC cells. As such, androgen- or NGF-challenging induces the assembly of AR/TrkA complex, which drives neuritogenesis in cultured PC12 cells [66] or mitogenesis and invasion in PC cells [68]. Thus, NGF or androgens may substitute each other’s in sustaining the proliferative or migratory phenotype of PC cells. As such, TrkA might undergo activation upon a local increase in androgen levels, while AR, which represents the hallmark of PC [87], can be activated by NGF. Noticeably, PC or their surrounding stromal cells frequently release androgens [88] or NGF [83]. By this way, NGF might foster the PC escape from the anti-androgen therapies to sustain by it-self PC aggressiveness. Precision strategies targeting the NGF signaling are then envisaged in PC patients.

Table 3 resumes a list of drugs challenging the drivers of NGF signaling in preclinical or clinical models of PC.

**Table 3:** Emerging interventions and drugs used in PC clinical trials or in PC preclinical models targeting Trk A, B, C receptors or activity

Type	Interventions	Study name	Condition/disease	Development stage	Refs or Study number
Pan Trk inhibitor and RTKs	CEP-701 (lestaurtinib)	Study of CEP-701 in Treatment of PC	PC	Phase 2	NCT00081601
TrkA,TrkB, TrkC, ROS1, ALK inhibitor	Entrectinib (RXDX-101)	STARTRK-1	PC and locally advanced or metastatic cancer confirmed to be positive for NTRK1, NTRK2, NTRK3, ROS1, or ALK molecular alterations	Phase 1	NCT02097810
Trk A selective inhibitor	VMD-928	Oral TrkA Inhibitor VMD-928 for Treatment of Advanced Adult Solid Tumors or Lymphoma	PC and advanced solid tumors or lymphoma	Phase I	NCT03556228
Pan-Trk kinase activity inhibitor	TQB3811	Phase I Clinical Study to Evaluate the Tolerability and Pharmacokinetics of TQB3811 Tablets in Patients With Advanced Malignant Tumors	advanced malignant solid tumor	Phase I	NCT05046847
Pan-TRK inhibitor	NOV1601(CHC2014)	A Phase 1, Open-label, Dose-escalation Study to Investigate the Safety, Tolerability, and Pharmacokinetics of NOV1601(CHC2014) in Adult Subjects With Solid Organ Malignancies	solid organ malignancies	Phase I	NCT04014257
ALK and pan-Trk inhibitor	TSR-011	A Phase I/IIa Open-Label, Dose Escalation and Cohort Expansion Trial of Oral TSR-011 in Patients With Advanced Solid Tumors	solid tumors	Phase I	NCT02048488
ROS1 and pan-Trk inhinitor	DS-6051b	A First-in-human Study to Evaluate the Safety, Tolerability and Pharmacokinetics of DS-6051b	solid tumors	Phase I	NCT02279433
Pan-Trk inhibitor	ONO-7579	ONTRK	solid tumors	Phase I	NCT03182257
Trk A inhibitor	GW441756		CRPC derived cells	Preclinical studies	[76]
Trk A constitutive Phosphorylation inhibitor	Altiratinib		PC-3 cells	Preclinical studies	[89]

*CRPC* metastatic castration resistant prostate cancer, *ROS1* receptor tyrosine kinase encoded by c-ros oncogene, *ALK* anaplastic lymphoma kinase

Overall, the findings so far obtained in PC cells strongly encourage the use of combinatorial therapies in clinical management of patients. It should be noticed that immune-cells surrounding PC cells express and release NGF [90]. Thus, the hypothesis that neurotrophins released by tumor associated-immune cells contribute to tumor innervation cannot be excluded. Combination of immunotherapies (anti-CTLA4, anti-PD1, anti-PDL1) with anti-neurogenic drugs to simultaneously counteract immune-escape and neurogenesis is, indeed, particularly attractive. Moreover, a neutralizing anti-NGF antibody, successfully employed in chronic inflammation and preclinical models of PC [91], might be used in combination with abiraterone or enzalutamide to inhibit both neurotrophic growth factor as well as AR signaling. This approach should reduce tumor survival as well as innervation and simultaneously alleviate the PC-related pain in patients with bone metastasis. Similar combinations might be envisaged in the treatment of brest cancer [52], further pointing to the connection between steroid endocrine system and NGF. In fact, the link we discovered between androgens and NGF is not unexpected. Estrogen replacement therapy affects the expression of NGFR in cholinergic neurons, thus playing a role in the cognitive functions associated with aging and neurodegenerative diseases [92]. Changes in circulating estrogen levels might contribute to the age-related changes in hippocampal levels of NGF [93]. Nasal administration of NGF improves the reproductive functions in mice exhibiting age-related hypogonadism and a reduction in androgen levels [94]. These and other findings previously discussed in this manuscript support a high degree of synergism between steroids and neurotrophins. Perturbation of this balance would enable the NGF signaling derangement in PC that express a plethora of SRs, including the estrogen receptors [95].

Another interesting aspect concerns the co-expression of TrkA and NGFR in PC. As before discussed in this section, NGF might induce mitogenesis or cell death upon TrkA or NGFR binding, respectively [73]. Thus, an oncogenic and an onco-suppressor role has been hypothesized for TrkA or NGFR, respectively [28, 70, 72–91]. These results, however, raise the issue of how and when the balance between TrkA and NGFR impinges on PC aggressiveness. NGF challenge of C4-2B cells, harboring both TrkA and NGFR, does not efficiently induce epithelial mesenchyme transition (EMT), while still mediating cell proliferation. By contrast, NGF robustly sustains EMT and mitogenesis in highly metastatic PC3 and DU145 cells, lacking NGFR and only expressing TrkA [76]. NGF likely induces in C4-2B cells a TrkA/NGFR dimer that albeit still able to engage and activate the circuits involved in mitogenesis (such as ERK and AKT), might be less efficacious in recruiting the signaling effectors leading to EMT, including Smad or non-Smad (FAK, Src tyrosine kinase, Grb2, mTOR) components. These findings further point to the onco-suppressor role of NGFR in CRPC cells and indicate that detection of TrkA or NGFR in PC specimens would offer predictive insights for patient’s stratification.

Beyond the mechanism(s) so far described, treatment-induced neuroendocrine differentiation of PC (tNEPC) is an intriguing challenge in diagnostic and clinical management of patients. tNEPC represents a process by which a subset of PC escapes the ADT and becomes more aggressive. These tumors often exhibit low or absent AR signaling, Rb and p53 loss, amplification of Myc-N, epigenetic changes and they are transcriptionally enriched for gene sets linked to neuritogenesis. All these features account for a highly aggressive phenotype and poor outcome [97]. Nevertheless, tNEPC markers are still far to be identified and targeted therapies are almost unavailable for tNEPC patients. It has been previously reported that PC cells overexpress Myc-N and exhibit low or absent AR activity after a prolonged ADT. These features lead to development of undifferentiated and invasive PC cells [98]. Similar findings have been subsequently reported by other groups [99]. Simultaneously, it has been shown that ZBTB46, a transcription factor stimulated by ADT, upregulates NGF, which, in turns, regulates tNEPC differentiation by physically interacting with the G-protein-coupled receptor, cholinergic receptor muscarinic 4 (CHRM4). Pharmacologic inhibition of NGF and knockdown experiments perturb the tNEPC differentiation mediated by CHRM4. Stimulation of CHRM4 is associated with ADT resistance and high levels of NGF in high-grade and small-cell NEPC patient samples. This study highlights the role of NGF in the development of NEPC and provides evidence that the NGF-CHRM4 axis represents a novel therapeutic target to impair NEPC progression [100]. Moreover, they significantly contribute to the understanding of unwanted effects caused by prolonged ADT in PC patients. In summary, from the reported findings it appears that ZBTB46 would predict the increase in NGF levels with the subsequent signaling derangement in PC patients, further indicating that NGF and their receptors are clinically actionable in NEPC [101].

## PC epithelial cells and tumor microenvironment (TME) as exchangers of NGF

The prostate gland develops from the urogenital sinus composed by epithelial (UGE) and mesenchymal (UGM) cells. Both the compartments are necessary for the prostate development, since if UGE and UGM components are separated and grafted alone into nude mice, neither of the two compartments can differentiate [102, 103]. Human prostate is indeed made up by the epithelial compartment, composed of exocrine glands and ductal structures, and the surrounding fibromuscular connective tissue stroma [104]. Thus, interactions between prostate epithelium and cellular constituents of the prostate stroma are crucial for organogenesis and the maintenance of normal organ function at maturity. During aging, molecular and structural changes might occur in TME, accounting for many pathological processes, including benign prostate hyperplasia, prostatitis and PC. Beyond the neoplastic epithelial cells, many stromal cells, dipped in the extracellular matrix (ECM), take part in the prostate tumor [105–107]. These cancer-associated stromal cells are mainly represented by myofibroblasts, smooth muscle cells, lymphocytes, adipocytes, endothelial cells, pericytes, macrophages, and mast cells. They promote cancer development and progression. In addition, epithelial cancer cells can functionally sculpt their microenvironment through the secretion of various cytokines, chemokines, and other factors [108]. The dialogue among PC cells and the surrounding stromal cells results in a liaison that fosters tumor growth, metabolic rewiring, stemness and metastatic events ([108]; Fig. 1).

Understanding the nature of this crosstalk would allow new therapeutic interventions that target TME components and ameliorate the patient’s outcome, even when the AR-based therapies fail. A lot of studies have demonstrated that PC and PC-TME cells are the most abundant source of biologically active NGF outside the nervous system [109]. Human prostate stromal cells express precursor forms of the NGF gene product [110, 111, 112, 113]. Consistent with the paracrine regulation of prostate tumor cell growth [109], the deriving mature forms exhibit biological activity and stimulate anchorage-independent growth of rat and human prostate epithelial cells expressing TrkA [113, 114]. Specific antibodies that neutralize NGF impair such paracrine-stimulated growth. Analysis of mRNA and DNA has shown that prostate stromal smooth muscle cells express NGF [115–117], and immunohistochemistry studies have shown that NGF is localized not only in the stromal compartment of normal and carcinoma samples, but also in benign prostatic hyperplasia and epithelial PC cells [71, 118]. Several PC-derived cell lines, representative of various degrees of malignancy, including LNCaP, DU145 and PC3 cells release abundant amounts of NGF, which might recruit prostate carcinoma-associated fibroblasts (CAFs) derived from human specimens [83]. Although it remains to establish whether CAFs express TrkA or NGFR, these findings support the idea of an intense paracrine-loop, by which stromal-derived NGF diffuses across the basement membrane to bind and activate TrkA or NGFR expressed by PC cells. In turn, NGF secreted by PC epithelial cells would activate the basic machinery leading to invasion of prostate CAFs, albeit other possibilities cannot be excluded, as discussed in the subsequent section of this review.

## The role of NGF in PC perineural invasion (PNI) and metastasis

**Fig. 2:**
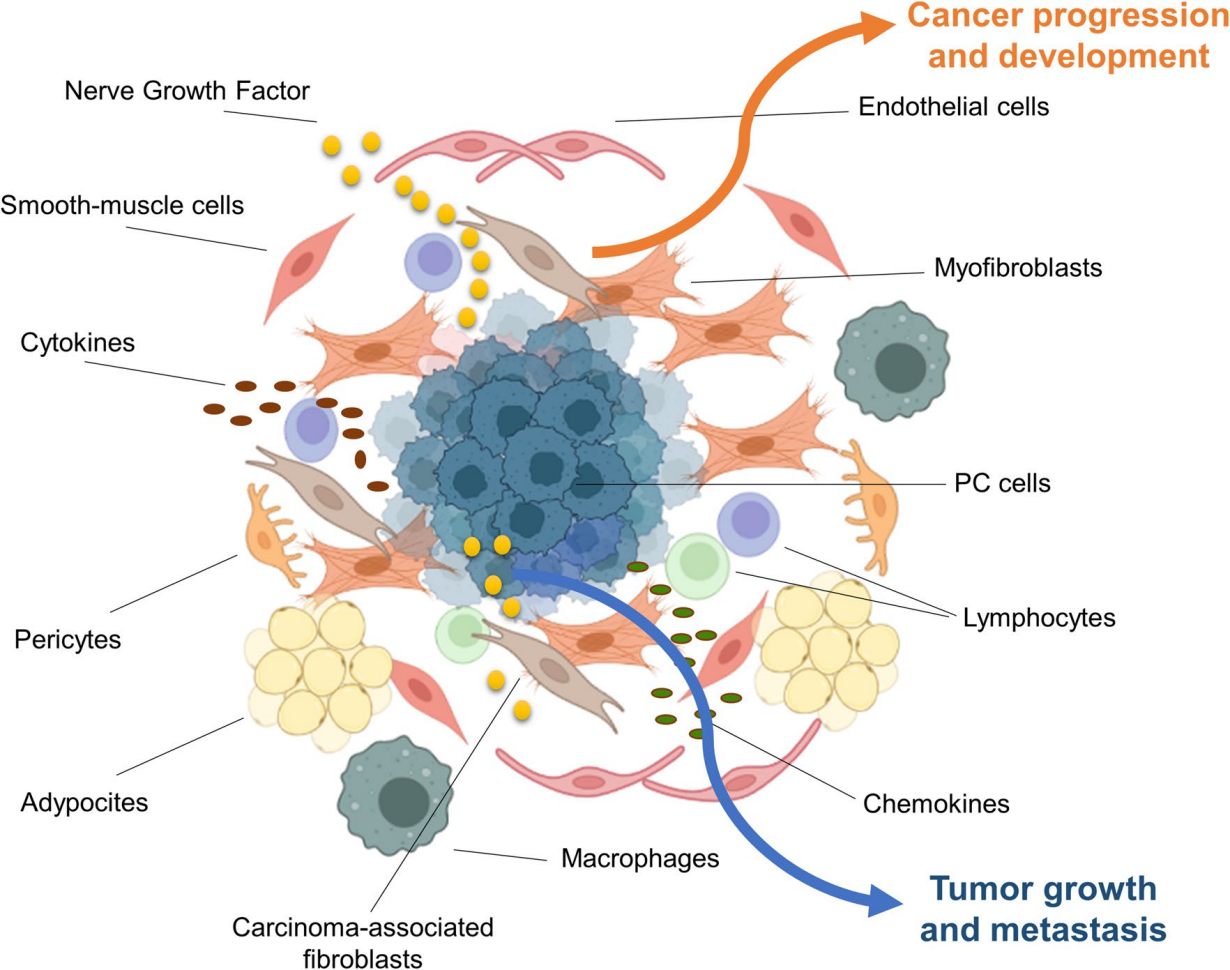
PC cells are surrounded by TME cells. Epithelial PC cells (blue) release cytokines, chemokines, and growth factors. Among them, NGF promotes tumor growth and metastasis. In tumor microenvironment, CAFs release significant amounts of NGF that binds TrkA or NGFR expressed by PC cells. In such a way, stromal cells can promote cancer progression and development

Metastatic disease is the leading cause of PC-associated death. PC cells undergo epithelial-mesenchymal transition (EMT), thereby acquiring a migratory phenotype and spreading as circulating tumor cells (CTCs; [119]. As shown in Fig. 2, the first site of PC spreading is represented by lymph nodes adjacent to the primary tumor [120]. Metastases to the liver and thorax then occur. By crossing the bone marrow stroma, PC cells might establish metastasis in bone [121]. In less than 1% of cases, PC might metastasize to the brain [122].

The expansion of micro-metastasis in bone involves a very dynamic process deriving from the interactions between PC cells, osteoblasts, and osteoclasts. Pathophysiology of osteolytic/osteoblastic metastatic bone disease in PC posits that metastatic tumor cells release humoral factors (osteoclast precursors, such as parathyroid hormone-related protein, interleukin-6) that stimulate osteoclastic recruitment and differentiation, while PC cells concomitantly produce soluble paracrine factors (TGF beta, IGF, bone morphogenetic protein), causing excessive osteoblast activation. Osteoclasts release growth factors, such as TGF beta that stimulate tumor-cell growth, perpetuating a vicious cycle of excessive bone resorption. In turn, activated osteoblasts release other growth factors that also stimulate tumor-cell growth, contributing to the perpetual cycle of abnormal bone formation. In this paracrine loop, the normal interplay between osteoclast and osteoblast activity is impaired, so that the imbalance in osteoblast/osteoclast activities might cause compensatory bone loss at skeletal sites distant from the sites of metastasis [123] [124] [125]. Noteworthy, in this intricate network FGFs deserve a particular mention, as their levels positively correlated with expression of TGF beta and the downstream signaling effectors [126]. FGFs are involved, indeed, in PC bone metastasis [127], and, among them, FGF9 mediates the formation of reactive stroma [128] as well as the osteoblastic progression of human PC cells in the bone of mice [129]. FGF pathway blockade could, hence, reduce the propensity of PC to metastasize and/or survive in bone.

**Fig. 3:**
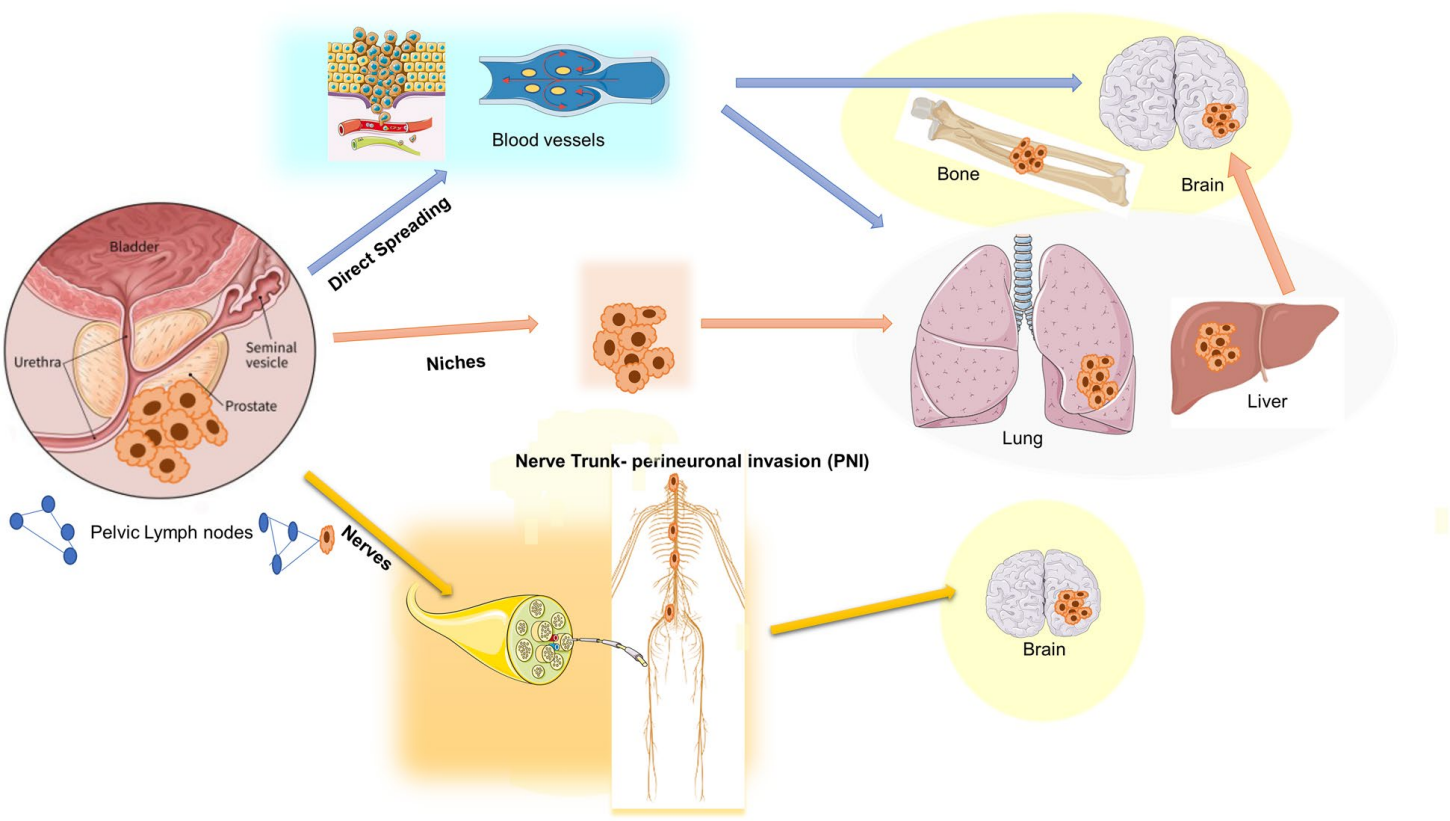
Firstly, PC cells spread to the pelvic lymph nodes. In addition, cells can migrate to the liver lung and thorax, or can establish in bone. In few cases, they metastasize to the brain, by directly spreading through the blood vessels or indirectly forming firstly “niches” in the liver or in the lung and then, disseminating into the brain. In some cases, PC cells might take advantage from the nerves surrounding the prostate capsule. Thus, they can spread through the perineural invasion (PNI) by following the nerve trunk

As before mentioned, PC cells can spread directly from the prostate to the brain through the blood stream. However, as shown in Fig. 2, they can indirectly reach the brain by first colonizing “niches” established in the liver or in the lung [130–132]. This step seems crucial, since the “soil” in which primary PC cells grow up is quite different from that of the brain. Fig. 3 As such, the cells would be unable to efficiently colonize in a new and different microenvironment. In liver and lung niches, however, the cells could remain in a dormant state for an indeterminate period to enjoy an environment conducive to their adaptation, until the conditions are favorable to generate cells genetically unstable and undifferentiated, with altered molecular signatures. At that stage, the cells might reach the brain microenvironment. Both the conditions, however, cause severe pain because of the involvement of tumor innervation. By contrast, the clinical pattern is quite divergent, as bone metastases are characterized by hypercalcemia and frequent fractures, while dissemination to the brain often induces edema and neurologic symptoms [133, 134].

Beyond the routes so far described, PC cells might become ‘neurotrophic’, as it frequently occurs in pancreatic ductal adenocarcinoma, gastric carcinoma, biliary tract tumor, head, neck, colorectal and cervical cancers [135]. The neurotrophic cancer cells might spread along nerves through the so-called ‘perineural invasion’ (PNI; [135] by following the nerve trunk. This process seems responsible for most extra-capsular spreading of PC cells [136]. Thus, metastatic cancers might acquire neuron-like signatures [137], including the expression of neuronal adhesion molecules, neurotransmitters, voltage-gated ion channels, neurotrophins and their cognate receptors [137]. The simultaneous expression of neurotrophin receptors on PC cells and the release of blood-borne ‘neuroactive’ substances [138] creates an autocrine- positive feedback loop, which fuels the tumor growth and guides the migration along the innervations. Such loop in PC biology is not unexpected, since the trophic influence of nerves play a fundamental role in morphological and functional changes of normal prostate. Hormonal regulation and autonomic nervous system control, indeed, the function and development of prostate gland, as the nerve input is involved in prostate epithelial differentiation from the first phases of development to the entire life span. Sympathetic and parasympathetic autonomic nerves supplied from the hypogastric plexus and the pelvic nerve, respectively, ensure the prostate innervation [139]. Nerves drill the prostate capsule and are distributed throughout the muscular layer, stroma, glandular cells and along the arteries. As such, they impact the surrounding structures through secreted neurotransmitters [140]. Interestingly, basal cells overexpress genes associated with neural development, neurogenesis, and axonal guidance, whereas luminal cells express genes involved in neural signal response and processing [141]. Of note, in PC-TME there is a high density of sympathetic and parasympathetic nervous fibers due to the high local levels of the precursor of NGF (proNGF; [59]). Cancer cells, endothelial cells, CAFs and immune cells surround the nerves in the TME. Thus, there is a reciprocal crosstalk between transformed cells, nerve fibers, blood vessels, ECM, reactive stroma and immune elements in TME, which actively contributes to cancer progression. Cancer cells release chemical messengers such as axon guidance molecules, NGF and vascular endothelial growth factor (VEGF) that by autocrine or paracrine loop modulate the activity of nerves when they are in the immediate microenvironment. On the other hand, when nerves lie more distally, exosomes represent a much more effient delivery system in promoting neo-neurogenesis, axogenesis, angiogenesis [142] and nerve infiltration of the tumour [143], given their ability to protect the cargo from the harsh TME pH, and to cover wide distances [9] Albeit the exosome-induced axonogenesis does not require NGF in PC [144], it has been recently shown that exosomes derived from PC patients with not favorable overall survival to Radium-223 are enriched of several oncogenic effectors, including NGF signaling components [145]. The different experimental and clinical conditions may account for the quite divergent reported findings.

Whatever the mechanism of axonogenesis promotion, PNI provides growth and survival advantage for PC cells in the perineural space where the cells acquire an increased metastatic potential [146, 146]. In this context, the excited nervous system releases neurotransmitters and neurotrophins, which trigger cancer aggressiveness. Thus, PNI provides microenvironmental factors that result in increased survival advantage for cancer cells in the perineural space. Of note, PNI increases the nerve growth in presence of PC cells [148]. Interestingly, the neurons density per ganglia increases in PC patients compared with controls and these patients exhibit a decreased and poor overall survival. This phenomenon might be due to a resident population of stem cells that, under the effect of local signals, give rise to new differentiated neurons [149]. It is still to be investigated if this type of neurotrophic PC develops in patients with predisposing higher nerve density or if cancer cells are responsible by themselves of the induction of neurogenesis, through a stemness program. Cancer stem cells (CSCs) can be regulated by the neuronal component of the TME and contribute to the aggressiveness of cancer [150]. For example, cutaneous sensory nerves, through nerve-induced hedgehog signaling, promote tumor formation in basal cell carcinomas arising from stem cells [150]. In addition, the neuronal input to the TME can indirectly affect CSCs through the activity of neurotrophins [151]. A cascade of released substances follows, so that neurons release neurotransmitters, which in turn promote the secretion of NGF and BDNF from cancer cells. This latter event facilitates the autocrine proliferation of CSCs. NGF, produced from proNGF cleavage, is the major promoter of axonogenesis (increase in nerve density) and neurogenesis (increased number of ganglion cell bodies) in neuronal, but also in PC cells. Axonogenesis and neurogenesis precede and facilitate the PNI at the initial stages of prostatic intraepithelial neoplasia (PIN) and the subsequent carcinogenic development [151]. However, their functional role in PIN is still unclear and an in depth understanding of the mechanisms responsible for PC cells/nerve interaction in cancer neurogenesis might greatly improve the development of new PC therapies. Although it is still unclear if axonogenesis/neurogenesis is concomitant to cancer progression or if it is a *conditio sine qua non*, it seems possible to assess that axonogenesis and neurogenesis take place within the TME by analyzing radical prostatectomies [152]. At this point, we attend to a well-organized process for which stem cells can differentiate into neurons [153], and innervation can promote CSCs within the tumour mass. This process is relevant from the clinical point of view, since the cancer stemness makes difficult a long-lasting effective therapy [154]. Given these premises and the complexity of this plot, exploration of cancer-related neurogenesis in PC pathogenesis and progression needs future in depth studies. Pharmacological intervention against neurotrophin signaling has the potential not only to directly target PC cells, but also to inhibit neurogenesis and its impact on PC progression and pain.

## The neurotrophic dependence of cancer cells in different types of tumors: a quick view

Although the neurotrophic addiction of PC has long been proven in preclinical models [155], clinical evidence is still scant. Nevertheless, a correlation between spinal cord injuries (SCI) and PC initiation and progression might be presumed, since patients affected by severe forms of myelopathy [156], or paralyzing injuries [157] exhibit a lower incidence of PC. The spinal cord lesions, associated with paralysis, partially or totally affect the prostate denervation and this scenario can be useful for understanding the impact of a functional denervation on prostate tumorigenesis. SCI patients show a decreased incidence of BPH and PC [158] and decreased levels of PSA [159], if compared with age-matched non-SCI patients. However, it is still to be explained if the reduced PC incidence in SCI patients is influenced by the lower neurotrophin’s supply to prostate. The neurotrophic dependence of cancer cells is also evident in other types of tumors. NGF promotes the innervation and proliferation of gastric and pancreatic cancerous epithelium and it is responsible for the tumor development [160, 161]. Accordingly, gastric or pancreas denervation reduces the tumor size as well as the incidence of gastric cancer [162], and slows the initiation and progression in mouse model of pancreatic adenocarcinoma [163]. Noteworthy, melanoma cells and epidermal melanocytic cells, derive from multipotent neural crest cells (NCCs) [164]. In the trunk, NCCs exit from the common site and give rise to neurons and glia of the peripheral nervous system and melanocytes in the skin [165]. This different fate is due to local and specific microenvironmental signals. Thus, melanoma cells share with NCCs the same differentiation markers involved in neural crest/melanocyte development and melanoma tumorigenesis [166]. Melanoma cells release significant levels of neurotrophins and express the Trk receptors and NGFR [167]. Particularly, NGFR expression seems involved in melanoma cell spreading to the brain. Normal brain cells, release NGF and NT-3 [62] that recruit melanoma cells to the brain to sustain their growth and survival [167]. A positive correlation between aggressiveness and innervation has been consistently reported in breast cancer (BC). TrkA and NGFR are expressed in various BC-derived cells that release abundant quantities of NGF [51] [52] [168]. As the surrounding TME and the associated nerve fibers also release NGF, an intricate dialogue among three different cell types occurs. In such a way, BC cells and TME components are recruited by the nerve fibers and migrate along them [51, 52]. This process correlates with an increase of relapse in TNBC patients, often exhibiting recurrence to CNS [169].

Despite the divergence between these cancers, it might be speculated that common mechanisms of interplay between different cell types are shared to facilitate tumor progression and metastatic spreading.

## Concluding remarks

Many human cancers, including PC, BC, melanoma as well as gastric, pancreatic and colon cancers might be considered neurotrophic cancers. In addition to the aforementioned features, these cancers might share other characteristics, thus opening unexpected and intriguing scenarios. As before discussed in this review, many evidence correlates the NGF signaling with the androgenic axis in the brain [66, 170–175], but also in some cancer types, including prostate [68] and colon [57]. The role of androgen/AR axis in neurotrophic cancers is supported by the following findings. Men are more likely than women to develop colon cancer, and hormone-replacement therapy in postmenopausal women reduces its incidence, suggesting a protective role for estrogen/ER in the development of this disease [173, 174]. Again, pancreatic cancer affects both men and women, but the mortality rate is higher in males as compared to females. A sex-related disparity in the incidence and prognosis of melanoma, with a higher survival rate for women has been reported [175, 176]. The AR expression is related to a poor prognosis in patients affected by melanoma with the acquisition of a metastatic phenotype [177]. By contrast, ERα expression is inversely related to the progression of the disease towards the more aggressive stages [178]. Furthermore, an increased risk of PC seems to be related to a greater risk of melanoma in bidirectional linkage [179], suggesting common signaling pathways for these types of cancer. As extensively discussed in this paper, a crosstalk between AR and Trk occurs in PC. A similar plot might control the aggressiveness of BC and melanoma that express AR at different degree. In sum, androgen/AR axis has an undeniable role in these cancers and future investigations are needed to clarify the intracrinology in these tumors and their related TME. These considerations suggest that a better understanding of the liaison between steroid- and neurotrophin-activated signaling pathways would offer new insights in the pathophysiology and therapeutic approach of these cancers.

As a greater understanding of nerve-cancer crosstalk and the neuro-immune axis emerges, new antineurogenic targets hold tremendous potential as novel opportunities for treating cancer. Thus, the study of mechanistic basis of cancer progression cannot neglect the nerve-cancer crosstalk and it might help identification of new therapeutic targets, thus allowing the repurposing of existing treatments or the identification of new drugs to be used in combo with chemo- or immune-therapies to slow or stop cancer progression.

## Data Availability

Not applicable.
